# Phthiriasis Pubis with Involvement of Axillae

**DOI:** 10.4269/ajtmh.22-0678

**Published:** 2023-02-20

**Authors:** Ze-Yu Luo, Jing-Fa Lu, Di-Qing Luo

**Affiliations:** 1 Department of Dermatology, Guangzhou Development District Hospital, Guanghzou, China;; 2 Department of Dermatology, The First Affiliated Hospital of Gannan Medical College, Ganzhou, China;; 3 Department of Dermatology, The East Division of The First Affiliated Hospital, Sun Yat-sen University, Guangzhou, China

**Figure 1.**
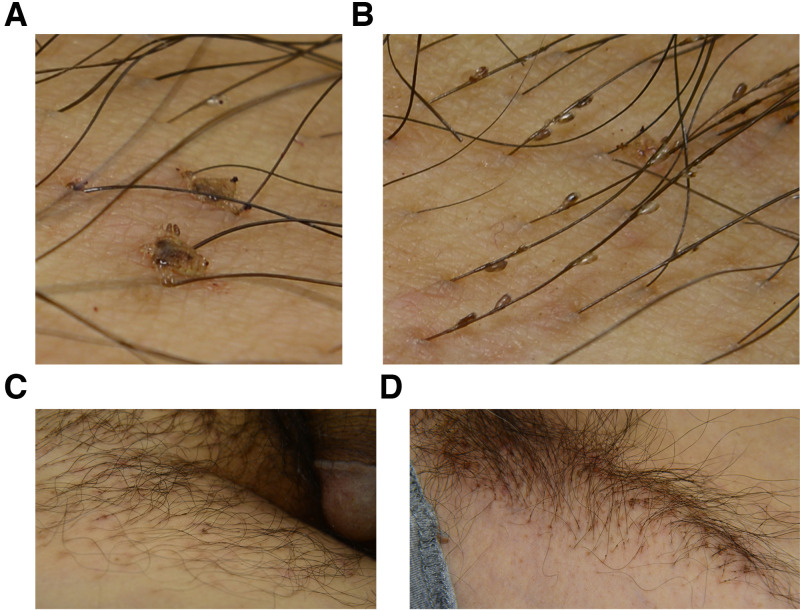
Adult lice on the skin that tightly clung to the pubic hairs (**A**), nits on the pubic hair shafts at an acute angle (**B**), and lice and nits on the pubis (**C**) and axillae (**D**).

**Figure 2.**
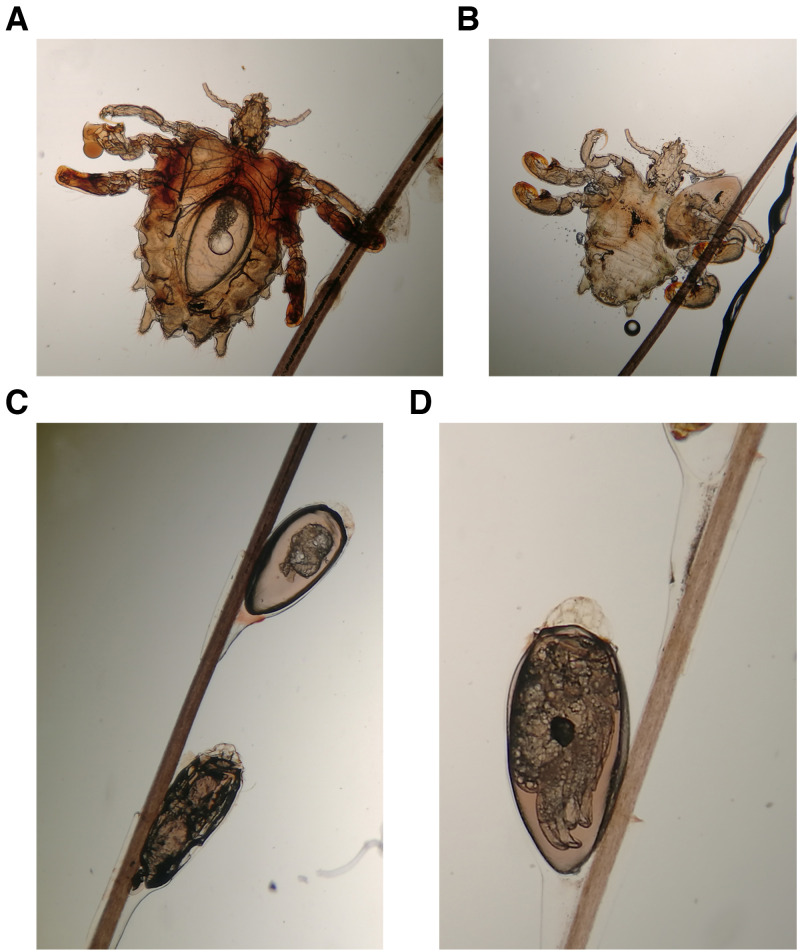
Female louse with nit inside (**A**), male louse with adjacent nit (**B**), nits on the hair shalt at an acute angle (**C**), and nits containing nymphs (**D**) under microscope.

A 28-year-old man was referred with a 2-month history of itching in the genital region that extended to the axillae 1 month earlier. The itching had poor response to oral antihistamines and topical steroid ointment. A clinical examination showed dozens of adult lice (Figure 1A) on the skin, grasping hairs tightly with claws, and hundreds of nits attached to the hair shafts at an acute angle (Figure 1B) on the pubis (Figure 1C) and axillae (Figure 1D). Female louse with egg inside (Figure 2A), male louse (Figure 2B), nits (Figure 2C), and nits containing nymph (Figure 2D) were detected under microscope. The lice were confirmed as *Pthirus pubis*. The patient was diagnosed as phthiriasis pubis with involvement of axillae. Both pubic and axillary hairs were completely removed, and the bedclothes, clothing, and fomites were treated with hot water. No lice and nits were detected at a 4-week follow-up.

Ectoparasites continue to be a common cause of skin disease worldwide. *Pthirus pubis*, the smallest of human lice, usually infests hairs in the pubic area and occasionally in areas heavily covered with body hair, including scalp, eyebrows, eyelashes, and axillae.^1^^,^^2^ The most common symptom of infection is pruritus. Its transmission relies on close contact.^1^ Increasing rates of body-hair removal might result in decreasing lice populations.^2^ The diagnosis of pediculosis pubis is based on the identification of live lice and/or viable nits.^1^ The present axillary pediculosis was considered to be transmitted from the pubic area by scratching.
